# Personalisierte Therapie des Phäochromozytoms

**DOI:** 10.1007/s00104-023-01988-6

**Published:** 2023-11-13

**Authors:** Nicolas Schlegel, Michael Meir, Joachim Reibetanz, Christian Markus, Armin Wiegering, Martin Fassnacht

**Affiliations:** 1 Klinik und Poliklinik für Allgemein‑, Viszeral‑, Transplantations‑, Gefäß- und Kinderchirurgie, Universitätsklinik Würzburg, Oberdürrbacher Str. 6, 97080 Würzburg, Deutschland; 2 Klinik und Poliklinik für Anästhesiologie, Intensivmedizin, Notfallmedizin und Schmerztherapie, Universitätsklinik Würzburg, Oberdürrbacher Str. 6, 97080 Würzburg, Deutschland; 3 Medizinische Klinik I, Lehrstuhl für Endokrinologie und Diabetologie, Universitätsklinik Würzburg, Oberdürrbacher Str. 6, 97080 Würzburg, Deutschland

**Keywords:** Nebennierentumoren, Metanephrine, Bildgebung, Genetische Testung, Präoperative α‑Blockade, Adrenal gland neoplasms, Metanephrine, Imaging, Genetic testing, Preoperative alpha blockade

## Abstract

**Hintergrund:**

Das Phäochromozytom ist eine seltene, aber schwerwiegende Erkrankung der Nebennieren. Ziel dieser Arbeit ist die Darstellung und Diskussion aktueller Entwicklungen zum diagnostischen und therapeutischen Vorgehen beim Phäochromozytom.

**Material und Methoden:**

Es wurde ein narrativer Übersichtsartikel auf Basis der aktuellsten Literatur erstellt.

**Ergebnisse und Diskussion:**

Der Anteil von Phäochromozytomen als Tumoren adrenalen Ursprungs liegt bei etwa 5 % der zufällig entdeckten Nebennierentumoren. Die klassische symptomatische Triade aus Kopfschmerzen, Schwitzen und Palpitationen tritt nur bei etwa 20 % der Patientinnen und Patienten auf, während fast alle Patientinnen und Patienten mindestens eines dieser Symptome aufweisen. Die Diagnostik umfasst im ersten Schritt den biochemischen Nachweis der freien Plasmametanephrine oder alternativ fraktionierter Metanephrine im 24-h-Sammelurin. Erst im zweiten Schritt erfolgt ein Schnittbildverfahren (Computer- oder Magnetresonanztomographie) zur Lokalisationsdiagnostik. Eine funktionelle Bildgebung ist ebenfalls sinnvoll, um eine Metastasierung präoperativ zu erkennen. Eine genetische Testung sollte immer im Verlauf der Behandlung angeboten werden, da 30–40 % der Phäochromozytome mit genetischen Veränderungen assoziiert sind. Das Dogma der präoperativen α‑Blockade wird durch aktuelle Daten zunehmend infrage gestellt und in den letzten Jahren kontrovers diskutiert. Die minimal-invasive Entfernung des Nebennierentumors ist Standard, wobei transabdominelle und retroperitoneoskopische laparoskopische Verfahren als gleichwertig anzusehen sind. Die Wahl des minimal-invasiven Verfahrens hängt von der Expertise und Erfahrung des Operateurs/der Operateurin ab und sollte in erster Linie daran angepasst werden. Postoperativ ist eine individualisierte und regelmäßige Nachsorge wichtig.

## Abstract (en)

**Background:**

Pheochromocytoma is a rare but severe disease of the adrenal glands. The aim of this study is to present and discuss recent developments in the diagnosis and treatment of pheochromocytoma.

**Material and methods:**

A narrative review article based on the most recent literature is presented.

**Results and discussion:**

The proportion of pheochromocytomas as tumors of adrenal origin is about 5% of incidentally discovered adrenal tumors. The classical symptomatic triad of headaches, sweating, and palpitations occurs in only about 20% of patients, while almost all patients show at least 1 of these symptoms. To diagnose pheochromocytoma, levels of free plasma metanephrines or alternatively, fractionated metanephrines in a 24‑h urine collection is required in a first step. In the second step an imaging procedure, computed tomography (CT) or magnetic resonance imaging (MRI), is performed to localize the adrenal tumor. Functional imaging is also recommended to preoperatively detect potential metastases. Genetic testing should always be offered during the course of treatment as 30–40% of pheochromocytomas are associated with genetic mutations. The dogma of preoperative alpha blockade is increasingly being questioned and has been controversially discussed in recent years. Minimally invasive removal of the adrenal tumor is the standard surgical procedure to cure patients with pheochromocytoma. The transabdominal and retroperitoneal laparoscopic approaches are considered equivalent. The choice of the minimally invasive procedure depends on the expertise and experience of the surgeon and should be tailored accordingly. Individualized and regular follow-up care is important after surgery.

## Hintergrund

Das Phäochromozytom ist eine seltene, aber schwerwiegende Erkrankung der Nebennieren, die durch eine übermäßige Synthese von Katecholaminen gekennzeichnet ist. Diese übermäßige Hormonproduktion kann zu einer Vielzahl klinischer Symptome führen, bei denen eine schwere Hypertonie, anfallsartige Kopfschmerzen und Palpitationen typische Leitsymptome sind. Der Großteil der Phäochromozytome verhält sich „benigne“. Die World Health Organization (WHO) führt Phäochromozytome inzwischen als Teilgruppe der Paragangliome auf, die alle als potenziell maligne gelten. In 10 % der Fälle treten Phäochromozytome als bilaterale Nebennierentumoren auf und sind in 30–40 % mit genetischen Veränderungen assoziiert. Die operative Entfernung des Phäochromozytoms stellt die einzige Möglichkeit einer kurativen Therapie dar. Verbesserungen des perioperativen Managements und der chirurgischen Technik haben die historisch hohen Mortalitätsraten auf 0–2,9 % gesenkt [1]. Aktuelle Entwicklungen des personalisierten therapeutischen Vorgehens beim Phäochromozytom werden nachfolgend dargestellt und diskutiert.

Entsprechend der WHO-Klassifikation für Paragangliome und Phäochromozytome sind Phäochromozytome als Tumoren adrenalen Ursprungs aus der Gruppe der Paragangliome anzusehen. Andere Paragangliome werden anhand ihrer anatomischen Lokalisation (z. B. abdominell, Kopf-Hals etc.) zugeordnet [2]. Mit einer Inzidenz 2–8/1 Mio. Einwohner/Jahr sind Paragangliome insgesamt seltene Tumoren [3]. Bei Patienten mit einem zufällig entdeckten Nebennierentumor (Inzidentalom) und arterieller Hypertonie liegt der Anteil von Phäochromozytomen bei etwa 5 % [4].

## Pathophysiologie von Phäochromozytomen

Grundsätzlich können Phäochromozytome sporadisch oder im Rahmen erblicher Syndrome auftreten. Bei etwa 30–40 % aller Patienten mit Phäochromozytomen lassen sich ursächliche pathogene Genvarianten nachweisen, die mit dem Auftreten dieser Tumoren in Verbindung gebracht werden. Die bekanntesten Veränderungen sind die multiple endokrine Neoplasie Typ 2 (MEN2), die von-Hippel-Lindau-Erkrankung sowie die Neurofibromatose 1 (Tab. 1). Hierbei ist das Auftreten bilateraler Phäochromozytome typisch. Pathophysiologisch kommt es durch Mutationen in spezifischen Genen wie *RET*, *VHL* oder den Succinat-Dehydrogenase(SDH)-Genen durch Störung verschiedener Signalwege zur gesteigerten und unkontrollierten Hormonsektion. Insgesamt zeigen Phäochromozytome damit eine erhöhte Expression und Aktivität von Enzymen, die an der Katecholaminbiosynthese beteiligt sind. Dazu gehören die Tyrosin-Hydroxylase (TH), welche Tyrosin in L‑Dopa umwandelt, und die Dopamin-β-Hydroxylase (DBH), die für die Umwandlung von Dopamin in Noradrenalin verantwortlich ist.

Die Hochregulation dieser Enzyme führt zu übermäßiger Katecholaminproduktion innerhalb der Tumorzellen. Zusätzlich kommt es zu einer vermehrten Sekretion dieser Hormone durch veränderten Vesikeltransport und Exozytose. Diese Veränderungen werden unter anderem durch Störung der zellulären Kalziumhomöostase sowie durch Störungen unterschiedlicher Signalwege des cAMP-Signalwegs, des Protein-Kinase-C-Signalwegs und des mitogenaktivierten Protein-Kinase(MAPK)Signalwegs induziert [1, 5].

**Table 1:** 

Gen	Keimbahnmutation	Somatische Mutation	Inzidenz bilaterale Phäochromozytome	Malignitätsrisiko/Metastasen
**Cluster 1: Genmutationen, die zu einer dysfunktionalen Veränderung der Hypoxieantwort führen**				
*VHL*	5–10 %	10 %	57 %	5 %
*EPAS1/HIF2a*	< 1 %	5–10 %	n/a	29 %
*EGLN1/2*	< 1 %	n/a	n/a	n/a
*SDH-x*	20–30 %	< 1 %	–	–
– *SDH-A*	< 5 %	–	4	12 %
– *SDH-B*	5–10 %	–	2 %	30–70 %
– *SDH-C*	< 5 %	–	< 1 %	< 1 %
– *SDH-D*	5–10 %	–	7 %	< 10 %
*SDHAF2*	< 1 %	–	–	–
*FH*	< 5 %	–	–	> 50 %
*MDH2*	< 1 %	–	–	?
*IDH1/2*	–	< 1 %	–	?
*SLC25A11*	< 1 %	–	–	Hoch?
*IDH3B*	< 1 %	–	–	?
*GOT2*	< 1 %	–	–	Hoch?
*DNMT3A*	< 1 %	–	–	?
*DSLT*	–	–	–	?
**Cluster 2: Mutationen von Genen, die zur Aktivierung von Kinasesignalwegen führen**				
*RET*	5 %	5 %	61 %	< 5 %
*NF1*	< 5 %	20–40 %	4 %	2–12 %
*TMEM127*	< 5 %	–	1 %	10 %
*MAX*	< 5 %	< 5 %	9 %	9–10 %
*H‑RAS*	–	5–10 %	–	–
*K1F1B*	< 1 %	20 %	–	–
*MEN1*	< 1 %	–	–	–

*n/a* nicht angegeben

## Herausforderungen bei der Beurteilung der Dignität von Phäochromozytomen

Eine Schwierigkeit ergibt sich dadurch, dass Phäochromozytome aufgrund histopathologischer oder molekularer Marker nicht eindeutig als gut- oder bösartig eingestuft werden können. Vielmehr zeichnet sich die Malignität durch invasives Wachstum oder durch das Vorhandensein von lokoregionären oder Fernmetastasen aus. Das rückt die Bedeutung einer regelmäßigen Nachsorge im Verlauf sowie die genetische Testung der Patienten in den Vordergrund, da Patientinnen und Patienten mit bestimmten Keimbahnmutationen (z. B. *SDH‑B*) ein deutlich erhöhtes Risiko eines malignen Wachstumsverhaltens zeigen [2].

Zur genaueren histopathologischen Beurteilung von Phäochromozytomen wurden zahlreiche unterschiedliche Scores entwickelt, von denen bisher keiner eine eindeutige prognostische Beurteilung des Präparates in Bezug auf das Risiko eines malignen Verhaltens ermöglicht. Dennoch erscheint es sinnvoll, diese anzuwenden, um eine individuelle Risikostratifizierung durchführen zu können. Im Würzburger Zentrum verwenden wir die Scoring-Systeme nach Thompson, das Grading System for Adrenal Pheochromocytoma (GAPP) nach Kimura, bei dem auch klinische Parameter zur Risikostratifizierung einfließen, sowie den Composite Pheochromocyteoma/Paraganglioma Prognostic Score (COPPS; [6–8]).

## Klinische Symptome und Diagnostik des Phäochromozytoms

In Abhängigkeit davon, welches Katecholamin übermäßig synthetisiert wird, können die Symptome unterschiedlich sein. Die klassische Triade aus anfallsartigen Kopfschmerzen, Schwitzen und Palpitationen tritt nur bei etwa 20 % der Patientinnen/Patienten auf, während fast alle Patientinnen/Patienten mindestens eines dieser Symptome aufweisen. Daher ist das klinische Bild nicht spezifisch und umfasst ein breites Spektrum mit Hypertonie, Tachykardie, Kopfschmerzen, Schwitzen, Blässe und Angst ([9]; Tab. 2). Durch die Hyperkatecholaminämie können als Folge der Hypertonie Herzinsuffizienz, Herzinfarkte, Apoplexien, spontane Blutungen oder dissezierende Aneurysmen auftreten [10].

**Table 2:** 

Quelle	Falhammar et al. [42]	Pourian et al. [43]
	Relative Häufigkeit (%)	Gepoolte Sensitivität (%)
Palpitationen	53	59,3
Hyperhydrosis	41	52,4
Kopfschmerzen	37	60,4
Müdigkeit	38	23,8
Orthostatische Symptome	27	57,5
Flush/Hitzegefühl	24	15,0
Übelkeit	22	21,2
Gewichtsverlust	16	*n*/a
Blässe	12	31,6
Asymptomatisch	9	*n*/a

Die diagnostische Sicherung eines klinischen Verdachts erfolgt zunächst durch die biochemische Diagnostik, im zweiten Schritt folgt die Lokalisationsdiagnostik [11]. Zur biochemischen Diagnostik erfolgt die Bestimmung der freien Plasmametanephrine oder alternativ der fraktionierten Metanephrinen im 24-h-Sammelurin. Insgesamt haben beide Bestimmungen eine hohe Spezifität, wobei die Sensitivität bei der Bestimmung der Metanephrine im Blut höher ist [3]. Hierbei ist es empfehlenswert, die Patienten vor der Blutentnahme ca. 30 min liegend ruhen zu lassen. Ist der Ruhewert mehr als 3fach über der Norm erhöht, so ist die Diagnose eindeutig. In unklaren oder grenzwertigen Fällen kann zusätzlich ein Clonidinhemmtest durchgeführt werden [12]. Es sollte auch darauf geachtet werden, dass potenziell interferierende Medikamente (z. B. Paracetamol, Monoaminooxidase(MAO)-Hemmer, trizyklische Antidepressiva) vor der Diagnostik abgesetzt werden.

Die funktionelle Bildgebung ermöglicht die frühzeitige Detektion von Metastasen

Zur Lokalisationsdiagnostik kommt ein Schnittbildverfahren (Computertomographie oder Magnetresonanztomographie) zum Einsatz. Entsprechend den Leitlinien ist eine zusätzliche funktionelle nuklearmedizinische Diagnostik nicht mehr zwingend notwendig, wobei dies in unserem Zentrum dennoch weiterhin häufig durchgeführt wird. Hintergrund ist, dass eine spezifische Diagnose einer malignen Erkrankung anhand biochemischer Marker präoperativ nicht möglich ist. Durch die mögliche Detektion von Metastasen in der funktionellen Bildgebung kann die therapeutische Gesamtkonzeption sowie die perioperative Vorbereitung angepasst werden. Als funktionelle Bildgebung kommt die Fluorodeoxyglucose-Positronenemissionstomographie/Computertomographie (FDG-PET/CT) oder das DOTA-TATE-PET/CT, das etwas sensitiver, aber deutlich spezifischer ist, infrage [13, 14]. In jedem Fall wird ein präoperatives Screening auf das Vorliegen von Metastasen mittels PET/CT empfohlen, wenn der Tumor > 5 cm ist, erhöhte Spiegel von 3‑Methoxytyramin (3MT) im Plasma vorliegen oder eine Keimbahnmutationen des *SDHB*-Gens bekannt ist [15].

**Figure 1:**
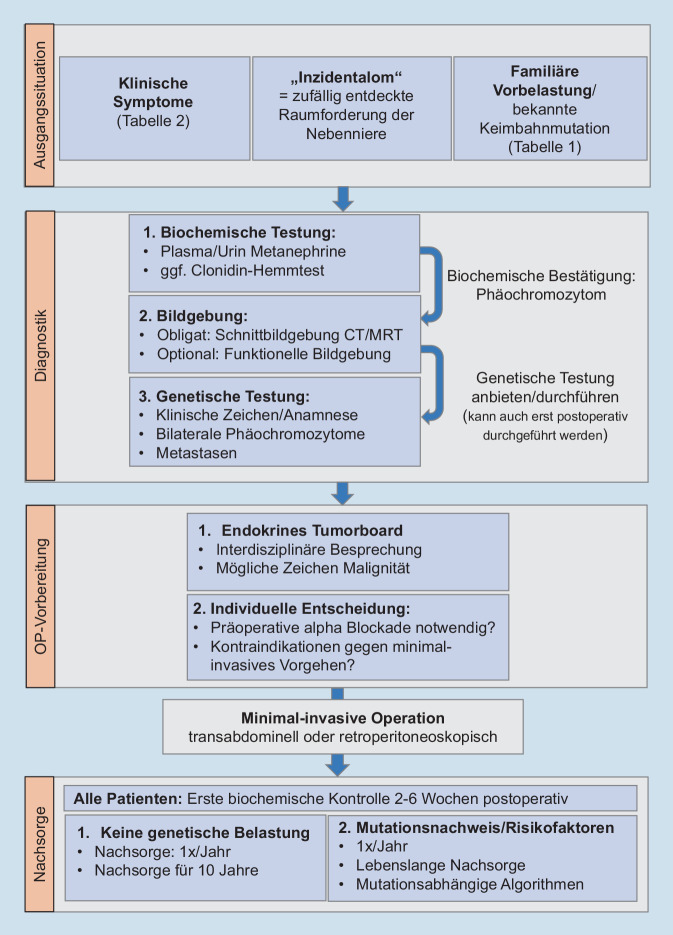

Bei negativer Familienanamnese und unilateraler Erkrankung ist bei älteren Patientinnen und Patienten der Nachweis einer Keimbahnmutation weniger wahrscheinlich. Dennoch wird heutzutage allen Patientinnen und Patienten mit Phäochromozytom eine humangenetische Diagnostik angeboten [16]. Die individuelle klinische Inaugenscheinnahme des Patienten/der Patientin ist in diesem Zusammenhang wichtig, um ggf. klinische Zeichen einer genetischen Erkrankung erkennen zu können (Abb. 1).

## Personalisiertes Vorgehen beim perioperativen Management

In den nationalen und internationalen Leitlinien [3] wird (noch) empfohlen, dass Patientinnen und Patienten mit Phäochromozytom eine präoperative α‑Blockade erhalten sollten. Ziele der präoperativen α‑Blockade sind die Vermeidung einer intraoperativen hämodynamischen Instabilität und die Vermeidung perioperativer Komplikationen.

Die α‑Blockade wird in der Regel etwa 10 bis 14 Tage vor der Operation begonnen. Aktuelle Studien zeigen dabei, dass es in der Wahl des α‑Blockers keine wesentlichen Unterschiede zwischen einem unselektiven Inhibitor wie Phenoxybenzamin oder den selektiven Inhibitoren wie Doxazosin gibt [17, 18]. Auch wenn es unter Phenoxybenzamin zu etwas geringeren Blutdruckschwankungen intraoperativ kommt, hat dies auf die Gesamtmorbidität der Patientin/des Patienten keinen Einfluss [19]. Bei Patientinnen und Patienten, die zusätzlich eine Tachykardie aufweisen, kann ein β‑Blocker ergänzt werden. Diese Medikation sollte jedoch frühestens nach 3‑ bis 4‑tägiger α‑Blockade begonnen werden, um eine hypertensive Krise durch die simultane Blockade der β_2_-Rezeptoren zu vermeiden. Zusätzlich oder alternativ können Ca^2+^-Antagonisten zur antihypertensiven Einstellung verabreicht werden. Es gibt eine erste randomisierte Studie die zeigt, dass eine präoperative Blutdruckeinstellung nur mit Kalziumantagonisten ohne α‑Blockade genauso effektiv in der Vermeidung perioperativer Komplikationen ist [20].

Allerdings zeigen trotz ausreichender α‑Blockade über 60 % der Patientinnen und Patienten intraoperativ Blutdruckschwankungen mit arteriellen Blutdruckspitzen von > 160 mm Hg, die allerdings nicht mit einer erhöhten kardiovaskulären Morbidität korrelierten [21, 22]. Zeitgleich werden bei Patientinnen und Patienten mit einer α‑Blockade präoperativ Nebenwirkungen wie orthostatische Dysregulation, vermehrte nasale Kongestion und reflektorische Tachykardien beobachtet [23].

Bei der präoperativen Blutdruckeinstellung sollte individualisiert vorgegangen werden

Vor diesem Hintergrund wird das Dogma der präoperativen α‑Blockade aktuell zunehmend infrage gestellt und in den letzten Jahren kontrovers diskutiert. In einer internationalen retrospektiven Multicenterstudie mit 1860 Patienten mit Phäochromozytom und Paragangliom zeigte sich, dass die kardiovaskuläre Komplikationsrate bei den 343 Patientinnen und Patienten, bei denen präoperativ keine α‑Blockade durchgeführt wurde, geringer war als bei den 1517 mit Blockade (0,9 vs. 5,9 %) [24]. Auch die Mortalität war in dieser Gruppe geringer (0,3 % vs. 0,5 %). Eine aktuelle Metaanalyse, in die 15 Studien mit über 3500 Patienten eingeschlossen wurden, zeigt im Vergleich zwischen Patientinnen und Patienten mit und ohne α‑Blockade keine signifikanten Unterschiede bezüglich perioperativer Blutdruckschwankungen, der Herzfrequenz und kardiovaskulärer Komplikationen. Jedoch war in der Gruppe der α‑blockierten Patienten postoperativ ein deutlich höherer Vasopressorbedarf zu beobachten [25]. Allerdings ist zu betonen, dass in diese Metaanalyse vor allem retrospektive Untersuchungen eingeflossen sind, die – genau wie die historischen Studien – einen Selektionsbias und methodische Probleme aufweisen. Es ist jedoch insgesamt eher anzunehmen, dass die Reduktion der perioperativen Mortalität auf < 1 % vor allem auf die Verbesserung der chirurgischen Technik mit minimal-invasivem Vorgehen ohne große Tumormanipulation, verbesserter präoperativer Diagnostik, Verbesserungen des hämodynamischen Monitorings und der Narkoseführung mit rasch wirksamen vasoaktiven Substanzen zurückzuführen ist und die präoperative Blutdruckeinstellung hierbei eine untergeordnete Rolle spielt [26]. Aufgrund der aktuellen Datenlage ist damit für die Patienten ein individualisiertes Vorgehen sinnvoll.

Wahrscheinlich ist die wichtigste Maßnahme, dass der Patient/die Patientin an einem großen spezialisierten Zentrum operiert wird, in dem alle beteiligten Abteilungen (Endokrinologie, Chirurgie und Anästhesie) mit den Besonderheiten dieses hormonaktiven Tumors vertraut sind. Ziel sollte aber auch sein, dass prospektiv Daten zum perioperativen Management gesammelt werden. In Expertenzentren erscheint es uns durchaus plausibel, auf eine α‑Blockade zu verzichten. An unserem Zentrum haben wir im Sommer 2022 nach intensiver interdisziplinärer Diskussion und Konsensusfindung diesen Weg eingeschlagen und behandeln präoperativ nur noch im Ausnahmefall mit α‑Blockade. Allerdings werden alle Patienten aufgeklärt, dass wir damit bewusst von internationalen Empfehlungen abweichen, und alle Fälle werden im ENSAT(European Network for the Study of Adrenal Tumours)-Register prospektiv erfasst, um eine Reevaluierung nach 50 Operationen zu ermöglichen. Zentren, die keine solchen Strukturen vorweisen können, sind wahrscheinlich besser beraten, vorerst weiterhin die α‑Blockade durchzuführen oder die Patienten in ein Zentrum mit entsprechender Expertise zu überweisen. Dies gilt besonders für Patienten mit großen, stark hormonproduzierenden Tumoren und schwer einstellbarem Bluthochdruck sowie für Patienten mit malignem Phäochromozytom.

## Die Wahl der Operationstechnik ist von der Expertise des Operateurs abhängig

Insgesamt sind Adrenalektomien seltene operative Eingriffe [27]. Es besteht laut Leitlinie der Konsens, dass die meisten Nebennierentumoren minimal-invasiv operiert werden sollten [28]. Die früher postulierte Grenzgröße der minimal-invasiven Chirurgie von 6–10 cm ist bei entsprechender Expertise vernachlässigbar. Wichtiger Aspekt ist hierbei, dass der Tumor ohne Kapselruptur in situ unabhängig von der Tumorgröße sicher zu entfernen ist [29]. Grundsätzlich besteht bei den minimal-invasiven Verfahren die Möglichkeit der transabdominellen und der retroperitoneoskopischen Adrenalektomie. Beide Verfahren können grundsätzlich auch roboterassistiert durchgeführt werden.

Das laparoskopische und retroperitoneoskopische Verfahren sind als gleichwertig anzusehen

Aus einer systematischen Übersichtsarbeit von 2016 geht hervor, dass die retroperitoneoskopische Adrenalektomie in Bezug auf die Operationszeit, postoperative Schmerzen und Rekonvaleszenz Vorteile bietet [30]. Eine Cochrane-Metaanalyse aus 5 Studien mit 244 Patientinnen und Patienten von 2018 konnte dies insgesamt nicht bestätigen [31]. Eine neuere vergleichende Metaanalyse zu diesem Thema mit 597 Patientinnen und Patienten in 6 Studien zeigte in Bezug auf die Komplikationsrate ebenfalls keinen Unterschied zwischen beiden Verfahren. Die retroperitoneoskopische Adrenalektomie war jedoch in dieser Arbeit mit einer kürzeren Operationszeit und geringeren Krankenhausverweildauer vergesellschaftet. Allerdings waren die Patientenkollektive nicht in allen Aspekten vergleichbar, da die Patientenkohorte, die retroperitoneoskopisch operiert wurde, insgesamt Patienten mit einem niedrigeren BMI und einer kleineren Tumorgröße umfasste [32]. Zusammenfassend sind auch auf Basis neuerer Daten das laparoskopische und retroperitoneoskopische Verfahren als gleichwertig anzusehen.

Im Vergleich zwischen roboterassistierter und konventionell-laparoskopischer Adrenalektomie ergab sich in einer Metaanalyse aus 4 Studien mit 386 Patientinnen und Patienten ein Vorteil für die roboterassistierte Adrenalektomie in Bezug auf hämodynamische Instabilität intraoperativ, den intraoperativen Blutverlust sowie die Krankenhausverweildauer [33]. Diese Vorteile werden in weiteren Metaanalysen bestätigt [34, 35]. Interessanterweise scheint das roboterassistierte Verfahren insgesamt oder zumindest beim transabdominellen Vorgehen sogar zu einer Verkürzung der Operationszeit zu führen [35].

Die Wahl des minimal-invasiven Verfahrens hängt zusammenfassend nach wie vor von der Expertise und Erfahrung des Operateurs/der Operateurin ab und sollte in erster Linie daran angepasst werden. Vergleichbar mit der Entwicklung in anderen Bereichen der Viszeralchirurgie zeichnet sich eine zunehmende Bedeutung der roboterassistierten Adrenalektomie ab.

## Rolle der parenchymerhaltenden Nebennierenresektion bei bilateralen Phäochromozytomen

Bei bilateralen Phäochromozytomen hat eine parenchymerhaltende Nebennierenresektion den Vorteil, dass hierbei idealerweise die Funktion der Nebennierenrinde erhalten bleibt. Hierzu muss mindestens ein Drittel des Nebennierenparenchyms einer Seite erhalten bleiben [36]. Dies war in einer Patientenkohorte von 66 Patientinnen und Patienten mit hereditärem Phäochromozytom nach bilateraler Resektion in 90 % der Fälle möglich [37] und deckt sich damit weitgehend mit den Ergebnissen einer größeren Registerstudie, in der der Funktionserhalt nach intendiertem Erhalt der Nebennierenrinde im Rahmen bilateraler Entfernungen von Phäochromozytomen über 70 % beschrieben wurde [38]. Der Vorteil des Funktionserhaltes muss jedoch grundsätzlich mit dem Risiko eines Tumorrezidivs abgewogen werden. Dieses ist in einer Registerstudie für einen medianen Zeitraum von 8 Jahren (4–13 Jahre) mit insgesamt 13 % angeben. Das Gesamtüberleben in dieser Studie ist dabei unabhängig von phäochromozytombedingten Komorbiditäten. Nur 5 % der 63 Verstorbenen in der Kohorte von insgesamt 625 Patientinnen und Patienten starben an den Folgen eines metastasierenden Phäochromozytoms. Im Gegensatz dazu entwickelte ein Drittel der Patienten Addison-Krisen oder ein iatrogenes Cushing-Syndrom [38].

Eine Metaanalyse aus 25 Studien mit insgesamt 1444 Patientinnen und Patienten kommt zu einem ähnlichen Ergebnis: Nach Erhalt der Nebennierenrinde im Rahmen einer bilateralen Entfernung von Phäochromozytomen besteht zwar prinzipiell ein erhöhtes Rezidivrisiko, jedoch ist das Risiko einer Metastasierung und die Gesamtsterblichkeit im Vergleich zur vollständigen bilateralen Adrenalektomie vergleichbar. Dagegen überwiegt der Vorteil eines Funktionserhaltes bei Belassen der Nebennierenrinde deutlich [39]. Eine aktuelle internationale Leitlinie zu Patientinnen und Patienten mit *SDH-B*-Mutation rät in dieser Patientengruppe allerdings dringend von einer organerhaltenden Operation ab, da hier das Malignitätsrisiko mit teilweise über 50 % doch beträchtlich ist. Sollte eine bilaterale Adrenalektomie durchgeführt werden, muss bereits perioperativ Hydrokortison verabreicht werden [1].

## Die Nachsorge nach Operation eines Phäochromozytoms ist individuell unterschiedlich

Für die Nachsorge von Phäochromozytomen wurde 2016 eine eigene Leitlinie verfasst [40]. Dort wird empfohlen, dass Patientinnen und Patienten ohne genetische Belastung für 10 Jahre einmal jährlich nachgesorgt werden sollten. Beim Nachweis einer Mutation oder anderer Risikofaktoren wie einem großen Tumor oder sehr jungem Patientenalter wird eine lebenslange Nachsorge empfohlen. Die erste postoperative Kontrolle findet nach etwa 2 bis 6 Wochen nach der Operation statt. Hierbei wird nachgewiesen, dass die präoperativ erhöhten Metanephrine nun normalisiert sind, was nochmals die komplette Resektion bestätigt. Spätestens zu diesem Zeitpunkt sollte dann jedem Patienten eine humangenetische Diagnostik angeboten werden, da diese – wie oben mehrfach aufgezeigt – Einfluss auf das Management hat. Je nach Mutation fokussiert die Nachsorge dann auch auf andere Erkrankungen (z. B. *RET*-Mutation: medulläres Schilddrüsenkarzinom und primärer Hyperparathyreoidismus; *SDHx*-Mutationen: hormoninaktive Paragangliome auch im Kopf-Hals-Bereich).

Um eine möglichst optimale Patientenversorgung zu gewährleisten, ist es wichtig, dass alle Patienten nach Adrenalektomie in einem interdisziplinären endokrinen Tumorboard vorgestellt werden und eine sichere Anbindung an den nachbehandelnden Endokrinologen erfolgt. Auf die Therapie des metastasierten Phäochromozytoms/Paraganglioms wird aus Platzgründen hier nicht eingegangen, sondern auf aktuelle Publikationen verwiesen [15, 16].

## Fazit für die Praxis

- Die Diagnostik beim Phäochromozytomen umfasst im ersten Schritt den biochemischen Nachweis der freien Plasmametanephrine oder alternativ fraktionierter Metanephrine im 24-h-Sammelurin, im zweiten Schritt ein Schnittbildverfahren (Computer- oder Magnetresonanztomographie) zur Lokalisationsdiagnostik. Zur Detektion von Metastasierung sollte präoperativ eine funktionelle Bildgebung erfolgen. Eine genetische Testung sollte im Verlauf der Behandlung angeboten werden.
- Die präoperative α‑Blockade wird zunehmen kontrovers diskutiert und sollte patientenindividuell erfolgen.
- Transabdominelle und retroperitoneoskopische laparoskopische Verfahren zur Entfernung des Nebennierentumors sind als gleichwertig anzusehen.
- Patienten ohne genetische Belastung sollten für 10 Jahre einmal jährlich nachgesorgt werden. Beim Nachweis einer Mutation oder anderer Risikofaktoren wird eine lebenslange Nachsorge empfohlen.
